# Spatiotemporal changes in net primary productivity before and after the development of unused land in the hilly areas of Hebei, China

**DOI:** 10.1371/journal.pone.0270010

**Published:** 2022-06-16

**Authors:** Li Zhao, Yaqian Chen, Xueyan Wang, Mengwei Su, Hao Xu, Pengtao Zhang

**Affiliations:** 1 Research Center for Local Culture and Rural Governance, Hebei Agricultural University, Baoding, Hebei Province, China; 2 School of Land and Resources, Hebei Agricultural University, Baoding, Hebei Province, China; 3 Taiyuan Design Research Institute for Coal Industry Group co. Ltd, Taiyuan, Shanxi Province, China; Jinan University, CHINA

## Abstract

Net primary productivity (NPP) plays an important role in the carbon cycle of an ecosystem. To explore the impact of unused land development on NPP, this study adopted an improved Carnegie Ames Stanford Approach (CASA) model to analyze the changes in NPP before and after the development of unused land in Tang County, Hebei Province, in 2000, 2007, and 2018. The results showed that, due to the changes in land use types from unused land, forestland, arable land with high NPP values to urban and rural residential land, traffic land with low NPP values, and the changes in precipitation and temperature, the NPP in the study area showed an overall trend of decreasing first and then rising from 2000 to 2018. Before the development of unused land in 2000, the total NPP was 38.45×10^10^ g C. After the development in 2007 and 2018, the total NPP was 36.44×10^10^ g C and 41.05×10^10^ g C, respectively. The NPP of each land type in 2018 was arable land (1046.18 g C m^-2^) > forestland (464.42 g C m^-2^) > unused land (356.34 g C m^-2^) > grassland (343.77 g C m^-2^) > waters (182.56 g C m^-2^) > urban and rural settlements (120.86 g C m^-2^) > traffic land (120.70 g C m^-2^). The distribution of NPP was generally high in the north and low in the south before and after development. NPP was mainly concentrated in the interval of 300 g C m^-2^ yr^-1^–400 g C m^-2^ yr^-1^, and the range of NPP change was mostly within 100 g C m^-2^. The influence of elevation, temperature and precipitation on the spatial distribution of NPP was significant. Elevation and precipitation were positively correlated with NPP, while temperature was negatively correlated with NPP. The increase in NPP mainly originated from the conversion of unused land to forestland and arable land. The loss of NPP was mainly due to the conversion from forestland with high vegetation productivity to a land use type with low vegetation productivity, such as the conversion from forestland to urban and rural residential land. The results can provide references for making reasonable land planning decisions and ecological environment construction.

## Introduction

Net primary productivity (NPP) refers to the total amount of organic matter converted from solar energy through photosynthesis minus remaining part after autotrophic respiration by plants during the same period, which is the primary source of food for heterotrophic organisms on Earth [1]. As an important part of material circulation and energy flow in the terrestrial ecosystem [2], NPP plays a fundamental role in the carbon source, sink and ecological processes of the ecosystem [3, 4]. At present, the models for estimating NPP can be broadly divided into climate-related statistical models [5], ecosystem ecological process models [6] and light energy utilization models [7], such as the Carnegie Ames Stanford Approach (CASA) model [8]. The statistical model is relatively simple, and the parameters are easy to obtain, but the error in the estimation results is large [9]. The ecological mechanism of the process model is clear, and the estimation result is accurate, but the model is complex, using many parameters that are difficult to obtain [10]. The CASA model fully considers the environmental conditions and ecological characteristics of vegetation and can directly obtain parameter values using remote sensing data, so it is widely used [11, 12].

With the rapid development of the economy, unused land has become an important resource, and the development of this land has become an important way to address land demand. However, the development of unused land will inevitably lead to a change in land use type, which will in turn directly affect the vegetation NPP [13]. Therefore, it is of great significance for regional ecological protection to discuss the impact of unused land development on NPP. At present, scholars are studying NPP on global and local scales [14, 15]. On a global scale, the study focuses on NPP of different vegetation types and its relationship with climate [16, 17]. On a local scale, NPP research mainly focuses on forests, watersheds and grasslands, and the influencing factors of NPP are attributed to the comprehensive effect of land use change, temperature, rainfall, topography and other factors [18–20]. [21] found that the average NPP increased by 52.4 gC m^-2^ yr^-1^ from 2000 to 2015 in Ximeng, and artificial woodland played a great role. [22]’s studies on Zhalong Wetlands showed that the annual average NPP of wetlands was 478.30 gC m^-2^ yr^-1^ from 2000 to 2017. [23] applied the CASA model to obtain an average NPP of grassland in northern China of 191.40 gC m^-2^ yr^-1^ and pointed out that the NPP of grassland was negatively correlated with temperature and significantly positively correlated with precipitation in northern China.

Generally, most of the existing literature focuses on the NPP of forests, grasslands, and watersheds in different regions of the world. The NPP of unused land before and after development in mountainous and hilly areas has rarely been discussed. However, a deeper understanding of these aspects can provide an important basis for future development management of unused land. Therefore, the main research objectives of this article are (1) to analyze the spatiotemporal evolution of NPP before and after the development of unused land in Tang County using the improved CASA model and (2) to identify the driving factors affecting NPP from the perspective of climate and land use types. In this way, we can better understand the impact of land use change on ecosystem service functions and provide a scientific basis for ecological environment protection and for the planning of land use patterns in the development of unused land.

## Materials and methods

### Study area description

**Fig 1:**
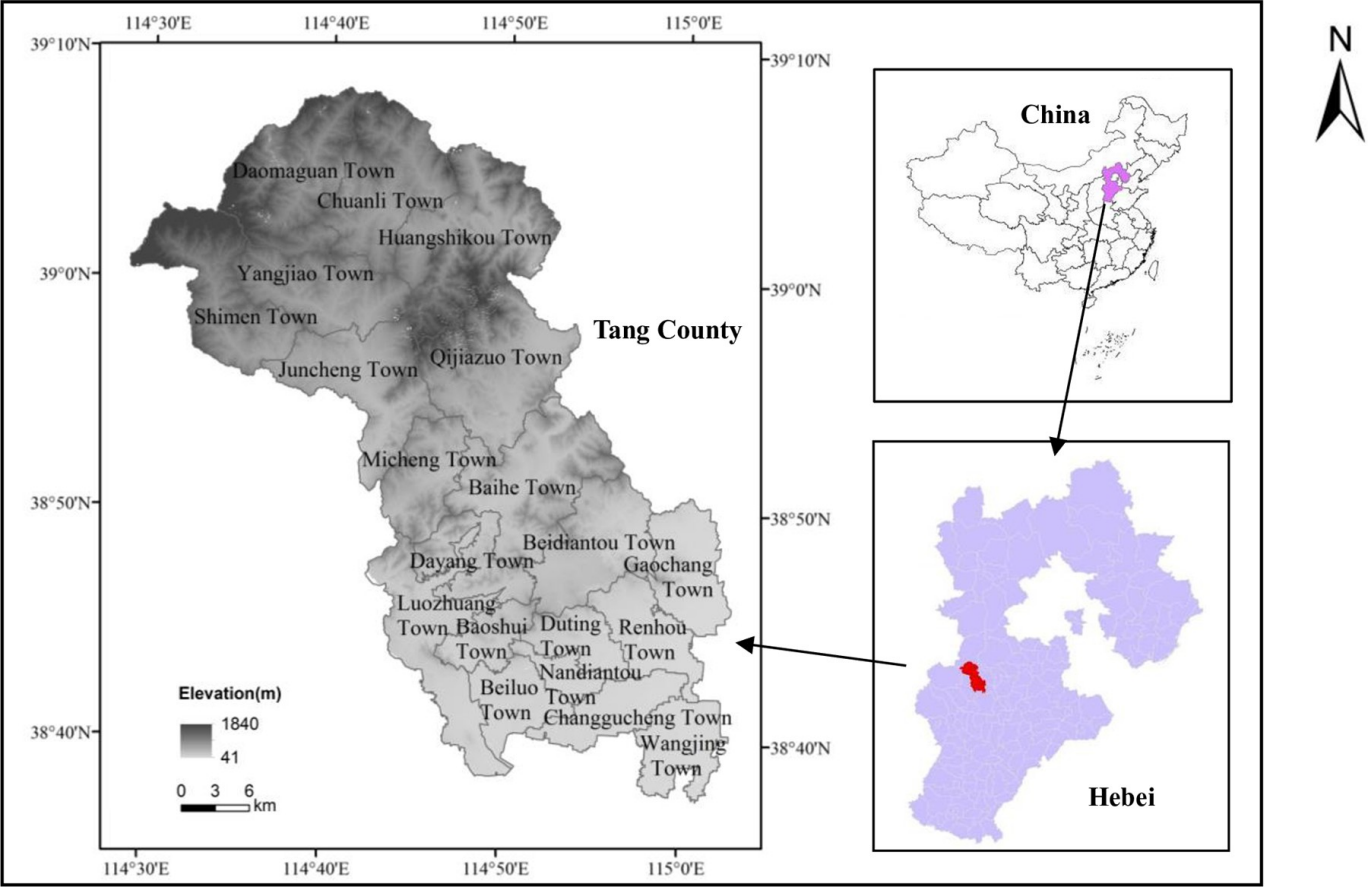
Location of the study area in Tang County district, Hebei Province, China.

Tang County, subordinate to the city of Baoding, is located in the middle of Hebei Province, China, 38°37’–39°09’ N, 114°27’–115°03’ E, with an elevation between 41 and 1840 m. Tang County is surrounded by Shunping County (east), Wangdu County, Dingzhou city (south), Quyang County and Fuping County (west), and Laiyuan County (north). Tang County has a warm temperate continental monsoon climate with four distinct seasons and a temperate climate. The regional average annual precipitation and evaporation are 575 mm and 1520.90 mm, respectively. The annual average sunshine hours are 2308.3 hours. The territory of the water system includes the Qingshui River and the Tang River. By the end of 2018, Tang County had jurisdiction over 7 towns, 13 townships and 345 administrative villages, with a total population of 602,600. The land area of Tang County is 1414.21 km^2^, the high-lying north-west, southeastern low. It belongs to a typical low hilly region, with an area of 1159.65 km^2^, accounting for 82% of the total land area of Tang County (Fig 1). In addition, Tang County is located in the Taihang Mountain area, which is an important mountain range and geographical boundary in eastern China, with a unique natural and cultural environment. It is not only an economically underdeveloped area but also a typical fragile ecological environment zone. It is also an important ecological barrier in Beijing, Tianjin and the North China Plain. With the promotion and implementation of targeted poverty alleviation policies and rural revitalization strategies, land use in this region will be greatly affected. The study of NPP changes and driving factors of unused land before and after development can provide an important basis for the management and direction of unused land development in this region and similar regions. This paper uses the unused land of Tang County in 2000 as the research object. The area of this study region was 90244.97 km^2^, accounting for 63.81% of the whole county area, which was the land use type with the largest proportion.

### Data and methods

#### Data source

In this study, remote sensing image data from 2000, 2007, and 2018 were downloaded from the Geospatial Data Cloud site, Computer Network Information Center, Chinese Academy of Sciences (http://www.gscloud.cn/). Different types of land use were classified, and three phases of land use maps before and after the development of unused land in Tang County were obtained. These were specifically divided into seven land types: arable land, forestland, grassland, urban and rural settlements, traffic land, waters, and unused land. The precision of classification was assessed using the Kappa coefficient test, with results showing coefficients above 0.80 for each phase of the department, which satisfies the requirement of classification accuracy. Topographic data, including elevation, slope and aspect, were derived from the Geospatial Data Cloud website (http://www.gscloud.cn/). The meteorological data were from the China meteorological data network (http://data.cma.cn/). The data included monthly precipitation, monthly average temperature, and monthly solar radiation. Normalized difference vegetation index (NDVI) data were obtained from the Chinese monthly vegetation index spatial distribution dataset of the Chinese Academy of Sciences Resource and Environmental Science and Data Center (http://www.resdc.cn/), including a total of 36 data periods in 2000, 2007, and 2018, with a uniform resolution of 30 m.

#### CASA model

The CASA model is a model of light energy utilization proposed by [24] and later improved upon by [25]. It has the advantages of a solid theoretical basis, relatively simple model structure, easy-to-obtain input parameters and strong universality, so it has been widely used in the estimation of vegetation NPP [26]. This study estimated the NPP of vegetation based on the improved CASA model of [25] and used the NPP software module V1.0 developed under the IDL software platform and its related static parameter simulation. The estimated NPP in the model can be represented by the absorbed photosynthetic active radiation (APAR) and the actual light energy utilization rate (ε):

NPP(x,t)=APAR(x,t)×ε(x,t) (1)

where *NPP*(*x*, *t*) represents the net primary productivity (g C m^-2^ yr^-1^), *APAR*(*x*, *t*) represents the absorbed photosynthetic active radiation by pixel *x* in month *t* (g C m^-2^ month^-1^), and *ɛ* (*x*, *t*) represents the actual light energy utilization rate of pixel *x* in month *t* (g C MJ^-1^). *APAR*(*x*, *t*) and *ɛ* (*x*, *t*) can be expressed by the following formulas:

APAR(x,t)=SQL(x,t)×FPAR(x,t)×0.5 (2)

where *SOL*(*x*, *t*) represents the total solar radiation amount (MJ m^-2^ month^-1^) at pixel *x* in month *t* and *FPAR*(*x*, *t*) is the absorption ratio of the vegetation layer to incident photosynthetic effective radiation. The constant 0.5 represents the proportion of solar effective radiation available to vegetation in the total solar radiation.


$$\varepsilon (x,\;t)=T_{\varepsilon 1}(x,\;T)\times T_{\varepsilon 2}(x,\;T)\times W_{\varepsilon }(x,\;t)\times \varepsilon_{max}$$ (3)

In Eq (3), *T*_*ɛ1*_ (*x*, *T*) and *T*_*ɛ2*_ (*x*, *T*) represent the stress at low and high temperatures, respectively, on the utilization rate of light energy. *W*_*ɛ*_ (*x*, *t*) is the influence coefficient of water stress, which reflects the influence of water conditions. *ɛ*_*max*_ is the maximum light energy rate (g C MJ^-1^) under ideal conditions.

#### Method of simple interpolation

The simple difference method uses the difference between images in the same area at different phases to represent NPP changes. The formula is:

$$D_{ij}=NPPt1_{ij}–NPPt2_{ij}$$ (4)

where *D*_*ij*_ represents the pixel difference value of Row *i* and Column *j*; *NPPt*1 _*ij*_ represents the NPP value of the pixel in Row *i*, Column *j* of phase *t1*; and *t1* and *t2* represent phases.

#### Correlation analysis

The correlation coefficient is a statistical index used to reflect the close correlation between variables. This article explored the correlation between NPP and climatic and topographic factors. The expression is as follows:

$$r=\sum_{i=1}^{n}\left(x_{i}-\bar{x})(y_{i}-\bar{y}\right)/\sqrt{\sum_{i=1}^{n}{\left(x_{i}-\bar{x}\right)}^{2}}\sqrt{\sum_{i=1}^{n}{\left(y_{i}-\bar{y}\right)}^{2}}$$ (5)

where *r* represents the correlation coefficient between NPP and the influencing factors, *x*_*i*_ represents the NPP value of the *i* grid, *y*_*i*_ is the factor value of the *i* grid, $\bar{x}$ represents the average value of NPP, $\bar{y}$ represents the average value of the influencing factors, and *n* represents the number of grids.

Then, we used the *t* test method to test the significance of the correlation coefficient. The calculation formula is as follows:

$$t=r\sqrt{n-2}/\sqrt{1-r^{2}}$$ (6)

where *r* represents the correlation coefficient and *n* represents the number of grids. We queried the *t* distribution table to judge the significance of the correlation coefficient.

## Results

### Interannual variation in NPP

We used the improved CASA model to calculate the NPP before and after unused land development (Table 1). The total NPP increased from 38.45×10^10^ g C before development in 2000 to 41.05×10^10^ g C after in 2018. The NPP in the study area showed a trend of first decreasing and then increasing as a whole. Compared with 2000, the total NPP increased by 2.6×10^10^ g C, which was mainly due to the increase in arable land and grassland. The total NPP can more profoundly reflect the total change caused by the conversion of land use types. The total NPP and its changes before and after the development of unused land were calculated (Table 1). From Table 1, it can be seen that arable land, forestland, grassland, urban and rural settlements, traffic land and water have increased the total NPP, while unused land has decreased it. Among them, the largest reduction in the total NPP was due to the large amount of development of unused land.

**Table 1:** Area of different land use types and total NPP and their changes before and after unused land development.

Type	Land area/hm^2^			Total NPP/×10^10^ g C				NPP variation/×10^10^ g C		
	2000	2007	2018	2000	2007		2018	2000–2007	2007–2018	2000–2018
**Arable land**		10309.08	13303.67			3.96	13.92	3.96	9.96	13.92
**Forestland**		12920.99	10534.99			6.22	4.89	6.21	-1.32	4.89
**Grassland**		14458.59	15867.12			5.58	5.45	6.21	-0.76	5.45
**Urban and rural settlements**		1829.56	4242.28			0.67	0.51	1.27	-0.76	0.51
**Traffic land**		625.04	200.44			0.22	0.02	0.22	-0.20	0.02
**Water**		473.16	1026.41			0.19	0.19	0.19	0	0.19
**Unused land**	90244.97	49628.55	45070.07	38.45		19.6	16.06	-19.82	-2.57	-22.39
**Total**	90244.97	90244.97	90244.97	38.45		36.44	41.05	-1.76	4.36	2.60

### Spatial variation analysis of NPP

**Fig 2:**
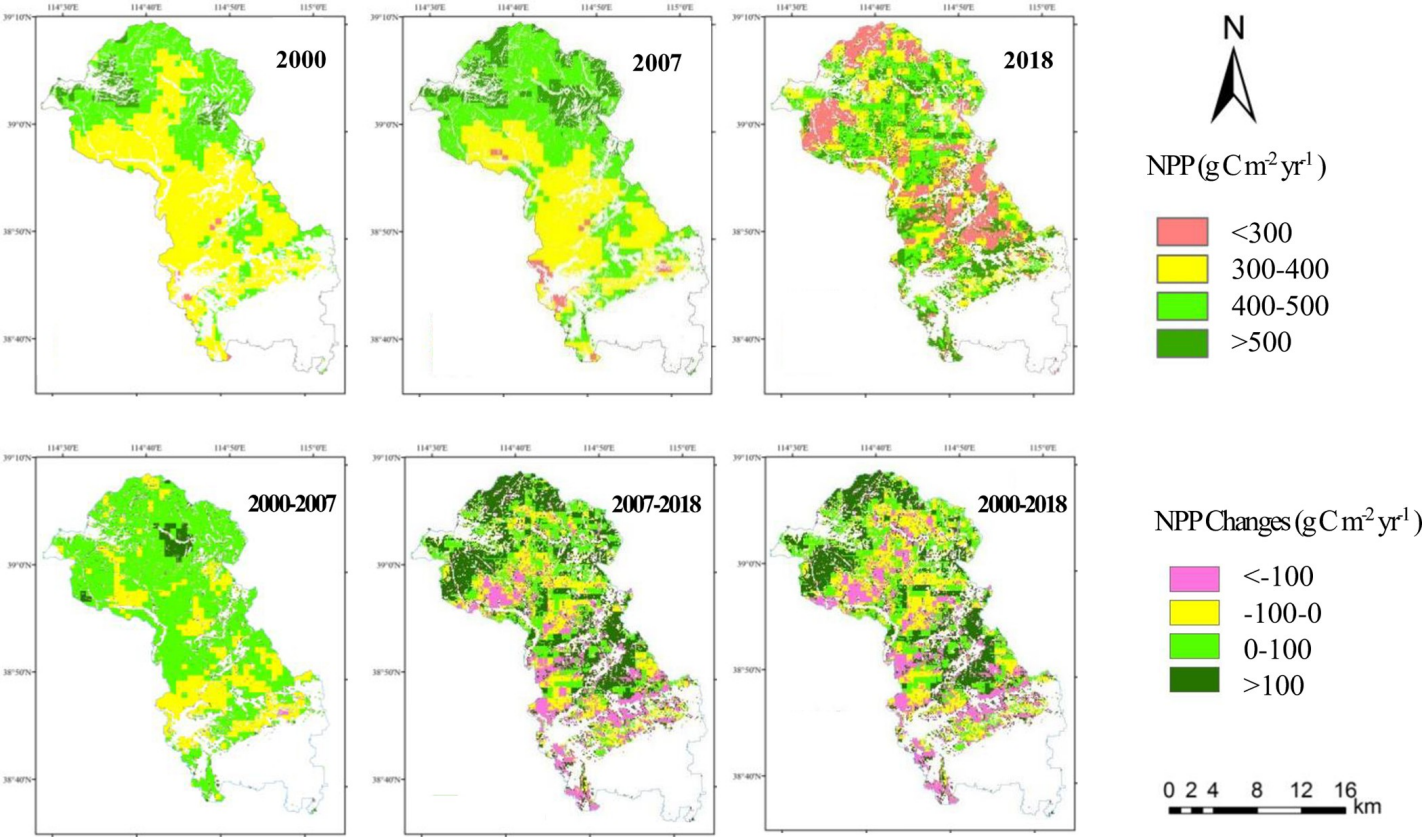
Spatial distribution of NPP for the Tang County region (2000, 2007 and 2014) and NPP changes for the periods 2000–2007, 2007–2018 and 2000–2018.

Based on the improved CASA model, the annual spatial distribution of NPP in Tang County before and after the development of unused land (2000) and after (2007 and 2018) was obtained. In general, the distribution was high in the north and low in the south. On the ArcGIS 10.2 platform, NPP in the research area from 2000 to 2018 was divided into four grades: <300, 300–400, 400–500, and >500 g C m^-2^, which correspond to low, medium, medium high and high values, respectively (Fig 2). NPP was mainly concentrated in 300 g C m-2–400 g C m^-2^, with a percentage of more than 40%, mostly in the central and northwestern parts of Tang County, followed by 400 g C m-2–500 g C m^-2^. The low- and high-value areas were less distributed, and the proportion of the two intervals was less than 30%. The low-value area was mainly distributed in southwestern Tang County, and the high-value area was mainly distributed in the northern area. This spatial distribution difference was mainly directly related to the distribution of land types.

From 2000 to 2007, the proportion of NPP in high-value areas was significantly reduced, with a reduction rate of 9.56%, which was reflected in the decrease in NPP in Huangshikou town and Daomaguan town, and the overall trend was downward. From 2007 to 2018, the proportion of low-value NPP areas increased, with an increase rate of 24.68%, mainly due to the decrease in NPP in central and northern Tang County. The high-value NPP areas were scattered, mainly in the middle and north of Tang County, and the overall NPP showed an upward trend.

The spatial variation in NPP before and after the development of unused land (2000–2018) was calculated by using the simple difference method (Fig 2). The variation range of NPP was mainly within 100 g C m^-2^. From 2000 to 2007, the NPP value decreased in most regions, mainly in the range of -100–0 g C m^-2^, accounting for 73.06% of the study area. From 2007 to 2018, the added value of NPP slightly exceeded the decreased value, and the increase range was 0–100 g C m^-2^. From 2007 to 2018, the decrease in NPP was less than the increase. Overall, compared with 2000, the area of increased NPP in 2018 was greater than that of decreased NPP, accounting for 43.18% of the total area. After the development of unused land, the average annual increase in NPP in the study area was mainly distributed in northwestern Tang County, including Yangjiao town, Qijiazuo town and Shimen town, while it showed a decreasing trend in the south and north-central parts of the study area.

### Analysis of influencing factors

#### Influence of climatic and topographic factors on NPP

**Fig 3:**
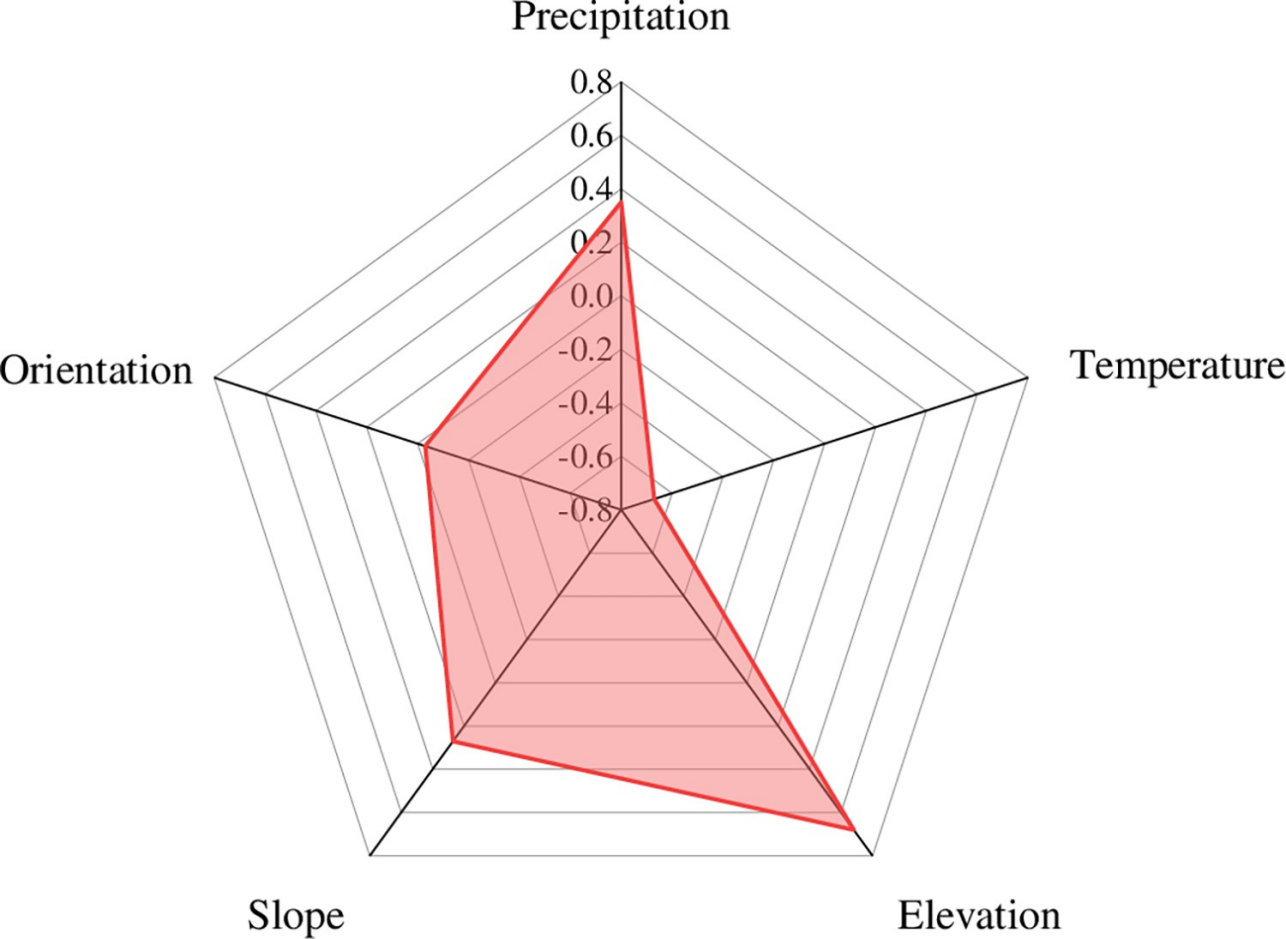
The correlation between average annual NPP and climatic topography in Tang County.

The spatial distribution of NPP was mainly affected by natural factors such as climate and topography. This paper selected precipitation and temperature as climatic factors and altitude, slope and aspect as topographic factors to analyze their impact on the current situation of NPP spatial distribution. The spatial distribution of NPP and climatic factors was expressed by the average value of three periods of data in 2000, 2007 and 2018. The correlation between climate and terrain factors and NPP was analyzed on the ArcGIS 10.2 platform (Fig 3), and the *t test* of the correlation coefficient showed a very significant correlation (P<0.01). It was concluded that the influence of each factor on NPP was different, and the precipitation, elevation and slope were positively correlated with NPP; that is, the NPP value increased with increasing index value. Temperature and slope direction were negatively correlated with NPP; that is, the NPP value decreased with increasing index value. Specifically, the correlation coefficients between NPP and precipitation, elevation and slope were 0.35, 0.68 and 0.27, respectively. Elevation had the greatest impact on NPP, followed by precipitation, and slope had a relatively small impact on NPP. The correlation coefficients of NPP with temperature and slope direction were -0.67 and -0.03, respectively, and slope direction had the least effect on NPP. Overall, elevation, temperature and precipitation had a great impact on NPP.

Based on the spatial analysis function of ArcGIS, principal component analysis was carried out on the five indicators of precipitation, temperature, elevation, slope, and aspect, and two principal components were extracted. The cumulative contribution rate was 81.82%, which reflected the initial information of all variables. Among them, the contribution rate of the first principal component was 47.78% and that of the second principal component was 34.04%. Table 2 shows the loading of each factor on the original index. From Table 2, it can be seen that the absolute loads of the first principal component (PC1) on precipitation and temperature were relatively large, indicating that these factors play an important role in this component. The second principal component (PC2) had a relatively large absolute load on the aspect, indicating that this factor had a significant effect on the second principal component, and the impact of climate on NPP was greater than that of terrain.

**Table 2:** The loading status of the factor to the original index.

Index	PC1	PC2
**Precipitation**	-0.5012	0.0239
**Temperature**	0.7961	-0.0337
**Elevation**	-0.2894	0.0085
**Slope**	-0.1719	0.0019
**Aspect**	0.0416	0.9991

#### Influence of land use type change on NPP

**Fig 4:**
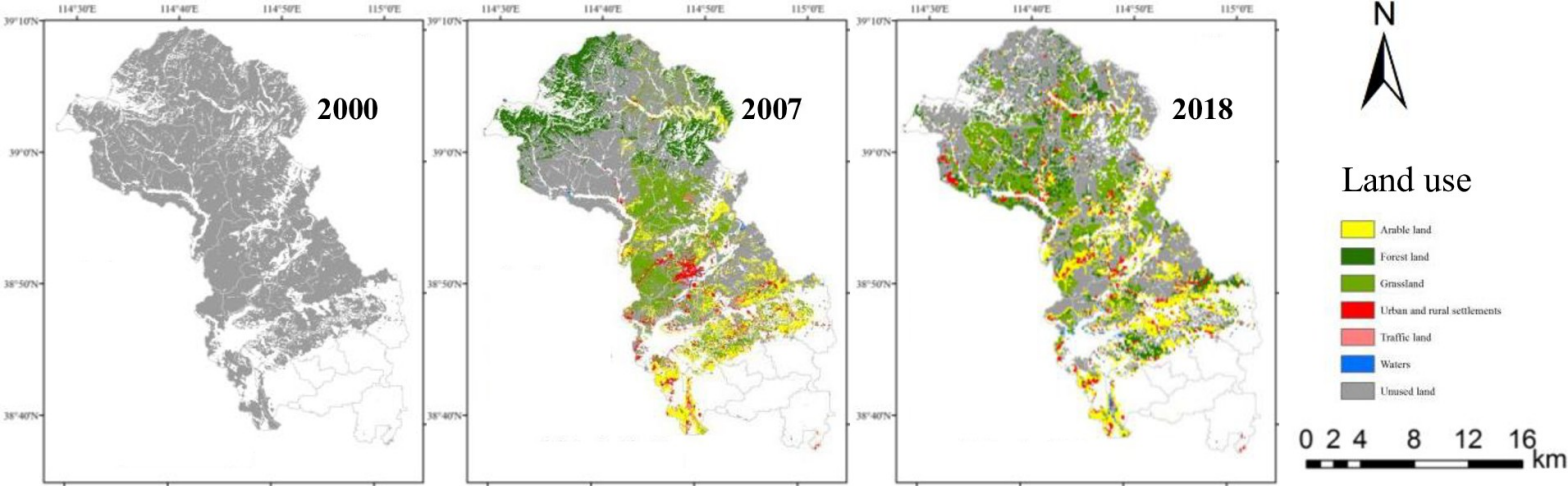
Land use before the development of unused land in 2000 and land use after the development of unused land in 2007 and 2018 in Tang County.

Before and after the development of the unused land (2000–2018), land cover in the study area changed significantly (Fig 4). After the development of unused land in 2000, the unused land area decreased greatly. From 2000 to 2007, the unused land area decreased by 46891.29 hm^2^, mainly developing into grassland, forestland and arable land, and the conversion rates were 17.72%, 14.01% and 11.34%, respectively. The urban and rural residential land, traffic land and water increased slightly to different degrees, with a total increase rate of 4.97%. From 2007 to 2018, the area of unused land continued to decrease, while the areas of grassland, forestland and water continued to increase. Among them, 54.99% of the unused land remained stable, 17.69% was converted to grassland, 12.68% to forestland, 17.07% to arable land and 1.68% to water. In general, from 2000 to 2018, after the development of unused land, the area of unused land decreased significantly, with a reduction rate of 66.30%. The area of other land use types increased, among which grassland, forestland and arable land areas increased the most.

The statistical analysis of NPP of various land use types showed that there were differences in the average annual NPP of different land-use types, and it can be seen that the conversion between different land types had different effects on regional carbon sink capacity.

**Fig 5:**
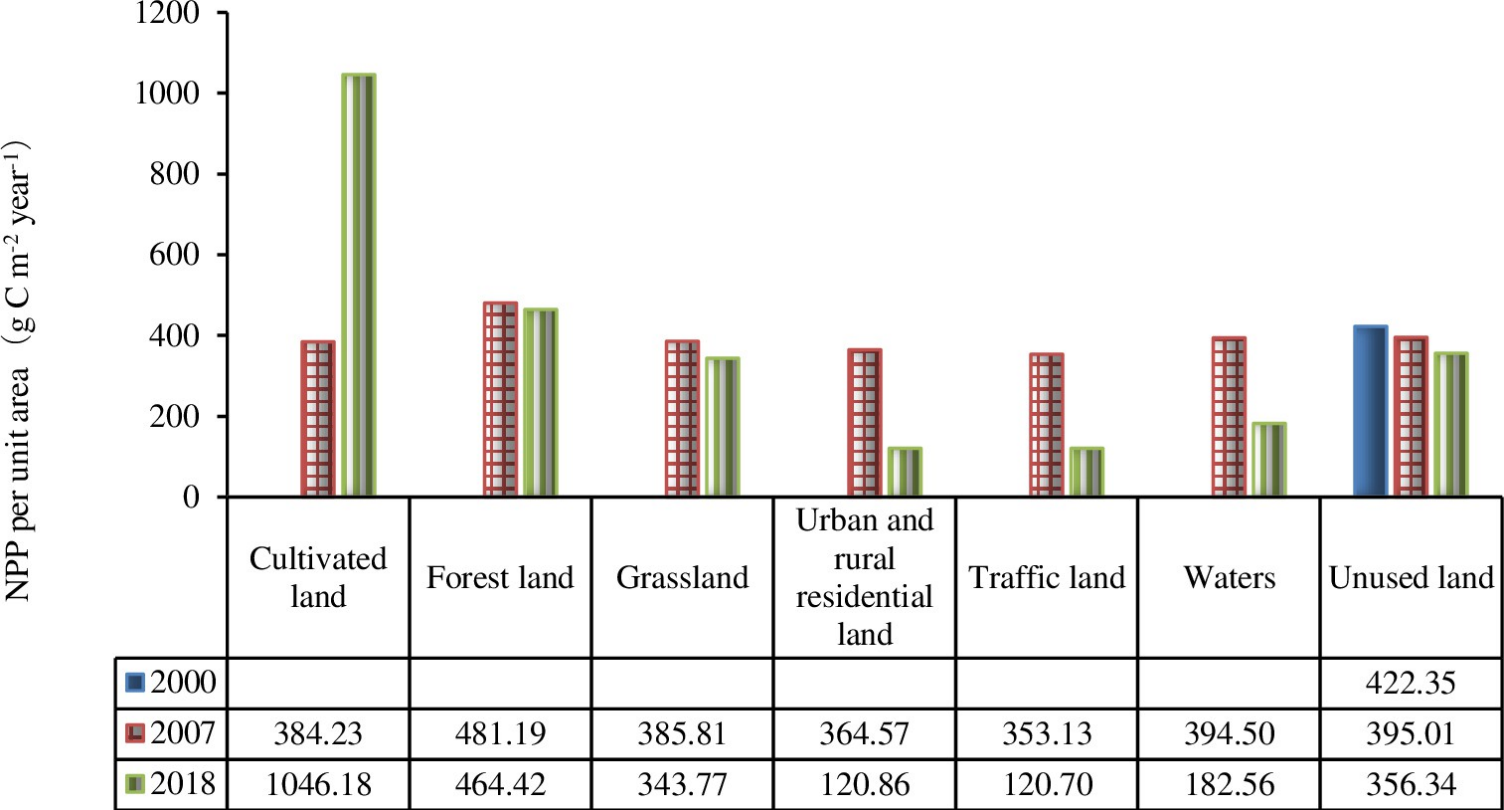
NPP values per unit area of different land use types in the study area from 2000 to 2018.

After the unused land was developed into different land use types, the NPP values changed. Before development in 2000, the NPP of the unused land was 422.35 gC m^-2^ yr^-1^, and after development in 2018, the NPP of arable land, forestland, grassland, urban and rural residential land, traffic land, water area and unused land were 1046.18 g C m^-2^ yr^-^1, 464.42 g C m^-2^ yr^-^1, 343.77 g C m^-2^ yr^-^1, 120.86 g C m^-2^ yr^-^1, 120.7 g C m^-2^ yr^-^1, 182.56 g C m^-2^ yr^-^1, and 356.34 g C m^-2^ yr^-1,^ respectively (Fig 5). From 2007 to 2018, the mean NPP of arable land increased significantly, with an added value of 661.95 g C m^-2^ yr^-1^. On the other hand, the mean NPP of urban and rural settlements, traffic land and waters decreased significantly, with decreases of 243.71 g C m^-2^ yr^-1^, 232.43 g C m^-2^ yr^-1^, and 211.94 g C m^-2^ yr^-1^, respectively.

Therefore, NPP first decreased and then increased from 2000 to 2018, which is directly related to the differences in climate conditions and land-type changes in 2000, 2007 and 2018. The NPP value per unit area of all land types, excluding forestland in 2007, was less than that of unused land in 2000 (Fig 5), and only 14.32% of the unused land was converted to forest, so the total NPP decreased in 2007 compared with 2000. The NPP value per unit area of cultivated land and forestland in 2018 was higher than that of unused land in 2000, and the area converted to cultivated land and forestland accounted for 26.42% of unused land in 2000. The NPP value per unit area of cultivated land was as high as 1046.18 g C m-2, which was 2.48 times that of unused land in 2000. This conversion of land is also the main reason for the increase in NPP in 2018 compared to 2000 and is also the reason why the NPP value in the north was relatively high and the NPP value in the south was relatively low. Due to the relatively high altitude in the north, the land types were mainly unused land, forestland and grassland with high NPP values. The terrain in the south was relatively flat, and the proportions of urban and rural residential, transportation and cultivated land use type were large, while the NPP value per unit area of urban and rural residential land and transportation land was relatively low.

## Discussion

### Spatiotemporal distribution of NPP

The study of NPP changes is of great significance to the carbon budget of the regional ecosystem. In this study, the improved CASA model was used to quantitatively evaluate the impact of unused land development on NPP. The research results showed that NPP was mainly concentrated in the range of 300–400 g C m^-2^ yr^-1^ before and after unused land development. In existing studies, [27] estimated NPP vegetation in the Taihang Mountain area and found that NPP in Tang County ranged from 200 g C m^-2^ yr^-1^ to 400 g C m^-2^ yr^-1^. [28] estimated the NPP of Hebei Province and concluded that the majority of NPP was between 200 g C m^-2^ yr^-^1 and 400 g C m^-2^ yr^-1^. [9] studied NPP vegetation in northern China and estimated that NPP in Baoding city ranged from 200 g C m^-2^ yr^-1^ to 600 g C m^-2^ yr^-1^. The findings of this paper are consistent with those of previous studies in similar areas.

In general, the NPP value of the study area was not high, indicating that there was no large increase in vegetation after the development of unused land or the low productivity of arable land, and residents paid little attention to greening, so ecological construction needs to be improved. After the development of the unused land, the areas where the average annual NPP increased in the study region were mainly distributed in northwestern Tang County, including Yangjiao town, Qijiazuo town and Shimen town. This is because the land improvement project in Tang County considered the influence of natural conditions such as topography and soil during the implementation. The northern part of Tang County is a mountainous and hilly area, which was mostly developed into forestland and grassland, so the NPP value of these areas was high. The overall NPP increased from 2007 to 2018, indicating that afforestation and agricultural development in Tang County have promoted the increase in NPP in recent years. Unused land type changed significantly after development, and the average arrangement size of NPP for each land use type was arable land (1046.18 g C m^-2^) > forestland (464.42 g C m^-2^) > unused land (356.34 g C m^-2^) > grassland (343.77 g C m^-2^) > waters (182.56 g C m^-2^) > urban and rural settlements (120.86 g C m^-2^) > traffic land (120.70 g C m^-2^) in 2018 (Fig 5). This is mostly consistent with the ranking of the average NPP of different vegetation types in Hubei Province by [18]. In addition, arable land and forestland have larger carbon fixation and oxygen release sources in this region, mainly because the light energy conversion rate of these two land types in this region was greater than that of other land types. This result is consistent with the research of [19, 29, 30]. [26] also showed that the NPP value of forestland was the highest, while the NPP value of land cover types without vegetation or low vegetation content was the lowest, which is consistent with the results of this paper. From 2000 to 2018, the average NPP of arable land and forestland increased significantly, showing that the arable land and forestland in the study area had conditions suitable for vegetation growth, and there may be a suitable tillage system and field management that make the arable land have higher farmland productivity. On the other hand, the traffic land, urban and rural settlement mean NPP significantly decreased, showing that the green area of construction land was reduced. This result from the conversion of land type is consistent with the study by [31], which mentioned that the increase in human activities is one of the reasons for the decrease in NPP.

### Effects of climate and terrain on NPP

Due to the comprehensive influence of land cover type, climate, terrain factors and other factors, the spatial distribution of NPP was uneven. Many studies [32–35] have shown that climate factors and human activities are the two main driving forces that influence NPP. The former changes environmental conditions in terms of the vegetation physiological structure and process, controlling the formation of NPP. Human activities, on the other hand, through production, life, or other activities, occupy NPP. From the perspective of climatic factors, moderate temperature can promote the growth of vegetation, while exceeding the optimum range of plants is not conducive to growth. An appropriate increase in precipitation can increase soil moisture, improve the soil water supply, increase the photosynthesis rate, and benefit vegetation growth. [20] studied the NPP of the tropical rainforest in Xishuangbanna and showed that precipitation had a threshold effect on NPP. The results of this paper showed that the correlation between precipitation and NPP was positive, while the correlation coefficient between temperature and NPP was negative; that is, the increase in temperature on NPP was mainly shown as inhibition. This is consistent with [27] analysis of the correlation between NPP and climate factors in the Taihang Mountain area. In this study, the correlation between temperature and NPP was -0.67, indicating that the NPP in the study area was more sensitive to the temperature response. [36] showed that in the Kalahari Desert, rising temperature leads to increased soil respiration, which causes a decrease in NPP. [30] stated that forest NPP increases with increasing temperature and precipitation. [32] reported that the annual NPP of tropical forests first increased and then decreased with increasing precipitation. The response of NPP to climate change showed spatiotemporal heterogeneity. The sensitivity of vegetation to climate change in different regions varies, and the limiting factors of vegetation growth were also distinct in different regions, which may be affected by the perennial climate characteristics and vegetation types in various research areas. From the perspective of terrain, elevation directly affects vegetation type distribution and temperature conditions, and slope plays an indirect role through slope erosion intensity. Therefore, the terrain also influences other environmental changes by controlling water, heat, and soil conditions, thereby affecting the vegetation pattern [37]. On the other hand, because places with higher altitudes have fewer human activities and most of the forestland is distributed, while places with lower altitudes are mostly construction and arable land and human factors significantly interfere in these areas, the NPP value is low, which is consistent with the discussion of [26] on the influence of altitude on NPP. In addition, through principal component analysis, this study concludes that precipitation, temperature and slope direction are the main indices affecting NPP in Tang County. Aspect will affect the temperature and humidity of the study area and the distribution of tree species, while precipitation and temperature will affect the growth of vegetation and crops. Therefore, the local governments and residents should fully consider the hydrothermal conditions required by vegetation and crops in the planning and planting process to improve the regional NPP.

### Impact of unused development on changes in NPP

The land use type changes after the development of unused land, and the impact of land use type on NPP is two-sided: on the one hand, land use type with higher vegetation productivity changes to land use type with lower vegetation productivity, such as construction land, resulting in the loss of NPP; on the other hand, the conversion of land use types with lower vegetation productivity, such as traffic land, urban and rural settlements, to land use types with higher vegetation productivity, such as forestland and cultivated land, brings about the increase of NPP. [38] also showed that land use change was the main reason behind America’s forest carbon sink, and the increase in forestland was the main reason for carbon accumulation. [19] found that the increase in forestland area caused by land use change also promoted the increase in NPP in south-central Chile. It can be seen from the change in the value of NPP of different land use types before and after the development of unused land that the net increase in NPP was caused by comprehensive factors such as climate and land use type, which was mainly reflected in the increase in NPP caused by the conversion of unused land to forestland. It is noteworthy that the development of unused land plays a positive role in the ecological construction of Tang County. [30] showed that the restoration of abandoned land or disturbed forest could improve NPP, and the main reason for the significant increase in NPP in the southeastern United States was the increase in forest cover. [39] also mentioned that the conversion of unused land to arable land promoted the increase in NPP. In the research of [12] on the NPP of Guangdong Province from 2000 to 2010, the area of unused land and the NPP value decreased, which is inconsistent with the research results of this paper. The reason is that although the unused land area decreased, the proportion of forestland and arable land area decreased even more, and the urban land area increased greatly, which led to the decline in NPP. In this study, the area of unused land was greatly reduced, which was mainly converted into forestland and arable land, thus increasing NPP. Combined with the actual situation of the study area and the results of this study, the unused land in Tang County will continue to decrease. Subject to the constraints of topographic conditions, economic development and the ecological environment, the development of unused land in Tang County will still tend to be mainly forestland and grassland in the north and construction land and arable land in the south. Under this background, the spatial distribution of NPP will continue the current distribution pattern; that is, the NPP in the north is higher than that in the south. At the same time, under the current national rural revitalization and ecological civilization construction strategy, more attention will be given to ecological environment construction, and the development of unused land will be more cautious. According to the current development trend, under the premise of reasonable land development and utilization, the NPP of the study area will tend to be stable or even rise in the future. Therefore, in future regional construction, we should pay attention not only to the suitability of land development but also to the greening of residential areas to improve the carbon fixation capacity of vegetation to create a good ecological environment. Attention should also be paid to the irrigation of cultivated land and fertilization measures to improve the productivity of arable land.

## Conclusions

Based on the improved CASA model, this study analyzed the interannual changes, spatial changes, and influencing factors of NPP before and after the development of unused land in Tang County (2000–2018) and obtained the following conclusions:

1. The total NPP in the study area presented an upward trend after an initial downward trend. In 2000, 2007 and 2018, the total NPP was 38.45×10^10^ g C, 36.44×10^10^ g C and 41.05×10^10^ g C, respectively. Compared with 2000, the total NPP increased by 2.6×10^10^ g C, which was mainly due to the increase in arable land and forest.
2. The spatial distribution of NPP in the study area tends to be uneven, with high distributions in the north and low distributions in the south. The NPP before and after the development of unused land was mainly concentrated in the interval of 300 g C m^-2^ yr-1–400 g C m^-2^ yr^-1^. The NPP variation range was mainly within 100 g C m^-2,^ and the average annual increase in NPP in the research area was mainly distributed in northwestern Tang County, including Yangjiao town, Qijiazuo town, and Huangshikou town, while it was decreasing in the south-central part of Tang County.
3. Elevation, temperature and precipitation in the study area had a significant influence on the spatial distribution of NPP. After land development, a large amount of unused land was converted under the combined effect of climate, topography and surface cover. The increase in NPP caused by the development of unused land in Tang County mainly came from the conversion of unused land and grassland to forestland and the conversion of construction land to grassland. Land use types with low vegetation productivity, such as the conversion from forestland to urban and rural residential land, result in a decline in vegetation carbon sequestration capacity.

In this paper, there are also some shortcomings, such as the fact that the relative contributions of climate factors and land use change to NPP are not considered. In future studies, these factors can be quantitatively evaluated to provide more references for land use planning and ecological construction.
